# Comprehensive analysis of transcriptome and microbiome in colorectal cancer with synchronous polyp patients

**DOI:** 10.3389/fcimb.2025.1547057

**Published:** 2025-04-17

**Authors:** Yubin Wang, Yongfeng Liu, Xiaoqiang Liu, Pengwei Xu, Mingjie Luo, Anle Huang, Zhijun Su

**Affiliations:** ^1^ Department of Gastroenterology, Quanzhou First Hospital Affiliated to Fujian Medical University, Quanzhou, China; ^2^ Department of Scientific Research Cooperation GeneMind Biosciences Company Limited, Shenzhen, China; ^3^ Department of Gastrointestinal Oncology Surgery, The First Affiliated Hospital of Xiamen University, School of Medicine, Xiamen University, Xiamen, Fujian, China; ^4^ Department of Infectious disease, Quanzhou First Hospital Affiliated to Fujian Medical University, Quanzhou, China

**Keywords:** colorectal cancer, gut microbiota, 16S rRNA sequencing, OTU-gene correlation, diagnostic markers

## Abstract

**Background:**

Colorectal cancer (CRC) is a prevalent and lethal malignancy, with the role of gut microbiota in its development still unclear. This study examines differences in gut microbiota between CRC patients and healthy controls and explores their association with host gene expression to identify potential diagnostic and therapeutic targets.

**Methods:**

Fecal samples from 10 CRC patients and 13 healthy controls were subjected to 16S rRNA sequencing. Transcriptome sequencing of tumor tissues, normal mucosa, and colorectal polyps from same 10 CRC patients was performed to identify differentially expressed genes (DEGs). Pearson correlation analysis was employed to associate operational taxonomic units (OTUs) with host gene expression.

**Results:**

β-diversity analysis showed significant differences in microbiota between CRC patients and controls (P < 0.01). LEfSe identified 38 distinct bacterial taxa, with genera such as *Bacteroides*, *Peptostreptococcus*, and *Parabacteroides* being enriched in CRC patients. Transcriptome analysis uncovered 1,026 DEGs. Notably, *TIMP1* and *BCAT1* were positively correlated (r > 0.76, P < 0.01) with pathogenic bacteria like *Fusobacterium nucleatum* and *Peptostreptococcus stomatis*. Tumor-related genes *TRPM4*, *MYBL2*, and *CDKN2A* were significantly upregulated and correlated with specific bacterial taxa.

**Conclusion:**

This study underscores the significant alterations in gut microbiota associated with CRC and reveals novel correlations between specific microbes and host gene expression, offering potential diagnostic markers and therapeutic targets for CRC.

## Introduction

Colorectal cancer (CRC) ranks as the third most common cancer globally and is the second leading cause of cancer-related mortality. Data from the National Center for Health Statistics on Cancer for the year 2024 indicate that there are approximately 1.93 million new cases of CRC and 940,000 CRC-related deaths worldwide annually (Siegel et al., 2024). The incidence of synchronous polyps in CRC patients varies from 14% to 48% (Arenas et al., 1997), with a predilection for the right or proximal colon. These polyps often manifest as multiple adenomatous polyps, some of which may demonstrate high-grade dysplasia or possess malignant potential (Demetriades et al., 2004). The presence of synchronous polyps can complicate treatment, potentially requiring more extensive surgical interventions and signaling an increased risk for the development of subsequent metachronous lesions (Li et al., 2019). Therefore, preoperative and postoperative colonoscopic assessments are crucial for the detection and management of synchronous polyps, enabling optimized treatment strategies and improved patient outcomes (Piñol et al., 2004).

The gut microbiota, an essential component of the human gastrointestinal tract, plays a key role in maintenance of host immunity, metabolism, and barrier functions (Valdes et al., 2018; Tilg et al., 2020). Recent studies have demonstrated a strong correlation between gut microbiota dysbiosis and the development and progression of CRC (Wirbel et al., 2019; Wong and Yu, 2019; Yachida et al., 2019). Despite this, research exploring the interplay between the gut microbiota and host gene expression is relatively scarce (Gopalakrishnan et al., 2018). Prior investigations have predominantly compared microbiota and gene expression profiles between colorectal polyps and cancer by examining samples from distinct patient cohorts (Choi et al., 2023; Zou et al., 2024). However, there is a paucity of research that specifically addresses CRC patients with synchronous polyps, creating an obvious gap in our understanding of the pathological mechanisms specific to this subtype of CRC.

To bridge the identified research gap, we conducted a comprehensive analysis by integrating microbiome and transcriptome data. This approach allowed us to systematically evaluate the differences in gut microbiota composition between CRC patients presenting with synchronous polyps and a cohorts of healthy controls. Furthermore, we investigated the correlations between specific microbial taxa and differentially expressed genes (DEGs) to uncover the potential roles of the gut microbiota in CRC pathogenesis. Our study aims to offer fresh insights into the mechanisms specific to this distinct patient group and to pinpoint potential therapeutic targets and intervention strategies tailored to this population.

## Methods

### Sample collection and processing

In this study, tumor tissues (CC), adjacent normal mucosa (NM), and synchronous colorectal polyp tissues (PP) were collected from 10 patients diagnosed with both CRC and synchronous polyps for transcriptomic sequencing. All tissue samples were immediately flash-frozen in liquid nitrogen upon collection and stored at -80°C until RNA extraction. Additionally, fecal samples were collected from the same 10 CRC patients and 13 healthy controls for gut microbiome analysis. The inclusion criteria were: (1) age between 18-80 years; (2) CRC group: diagnosis of colorectal cancer confirmed by colonoscopy and pathology; control group: no abnormalities detected in colonoscopy; and (3) voluntary participation with signed informed consent. Exclusion criteria included: (1) familial colorectal cancer or familial polyposis; (2) history of diabetes; (3) use of antibiotics or probiotics in the past three months; (4) symptoms of infection within the last week; and (5) presence of other intestinal diseases. The study protocol was approved by the Ethics Committee of Quanzhou First Hospital (Ethics Approval Number: [2024] K189.

### 16S rRNA sequencing and analysis

Genomic DNA was meticulously extracted employing the CTAB/SDS protocol. The quantification and assessment of DNA purity were conducted using 1% agarose gel electrophoresis. Subsequently, the DNA was adjusted to a uniform concentration of 1 ng/μl using sterile water to standardize subsequent procedures. To construct sequencing libraries, the V3-V4 hypervariable regions of the 16S rRNA genes were selectively amplified. This was achieved using the NEBNext^®^ Ultra™ DNA Library Prep Kit for Illumina^®^ (New England Biolabs, Ipswich, MA, USA), adhering closely to the manufacturer’s guidelines. Unique index codes were incorporated to facilitate sample tracking. Post-quality control (QC), the libraries were subjected to high-throughput sequencing on an Illumina MiSeq platform, yielding 250 bp paired-end (PE) reads.

The sequences underwent stringent quality filtering using Trimmomatic V0.33 (Bolger et al., 2014), ensuring that only high-quality clean reads were retained for further analysis. Sequence assembly was performed using FLASH (v1.2.11), and the resulting contigs were clustered into operational taxonomic units (OTUs) at a 97% similarity threshold using the VSEARCH clustering algorithm (Rognes et al., 2016). For each OTU, a representative sequence was selected for taxonomic annotation. Taxonomic classification was conducted using QIIME2 (2019.4) with a confidence threshold of 0.7, leveraging the comprehensive Silva (Release 132) database for accurate species identification (Quast et al., 2013). Diversity indices, specifically the Shannon index, were calculated to assess alpha diversity for each sample. Beta diversity was analyzed using non-metric multidimensional scaling (NMDS) to visualize community structure differences between samples. To identify differentially abundant features, linear discriminant analysis effect size (LEfSe) was applied. The analysis was conducted using the Python LEfSe package, with a Wilcoxon p-value threshold set at 0.05 and a logarithmic LDA score cutoff at 2.0 to ensure statistical robustness. Finally, the results were visualized using bar plots and heatmaps generated with the R package v3.4.1, providing a clear and concise graphical representation of the data.

### Transcriptome sequencing and analysis

Total RNA extraction from tumor tissue, normal mucosa, and colorectal polyps was conducted according to previously established protocols (Liu et al., 2021). Subsequently, RNA-sequencing (RNA-seq) libraries were meticulously prepared using the AHTS Universal V8 RNA-seq Library Prep Kit for Illumina (Vazyme, China), strictly adhering to the manufacturer’s instructions. These libraries were then subjected to sequencing on the SURFSeq 5000 platform (GeneMind Biosciences LTD., China) employing a 150-cycle paired-end high-output sequencing protocol. Post-sequencing reads were meticulously filtered to remove adapters, poly-N sequences, and low-quality reads. The cleaned reads were subsequently aligned to the human reference genome (GRCh38/hg38) to ensure accurate mapping.

To explore the relationship between microbial community composition and host gene expression, the Pearson correlation coefficient was employed to assess the correlation between OTU abundance and differential gene expression levels for each OTU-gene pair across all 10 patients. To enhance computational efficiency and precision, OTUs present in fewer than 2 patients were excluded, resulting in a refined dataset of 298 OTUs that accounted for 98% of the initial abundance. DEG analysis was performed using DESeq2 to compare two groups per patient: normal mucosa versus colorectal tumor tissue (NM-VS-CC) and polyp tissue versus colorectal tumor tissue (PP-VS-CC) (Love et al., 2014). Genes were considered DEGs if the fold change (|log2FC|) between groups was >=1 and the differences were statistically significant (P-adjust <=0.05).

In correlating differential gene expression with OTU abundance, only the intersection genes of the two DEG sets were selected for analysis. This approach was taken to quantify the correlation between the host transcriptome and the microbiome. The Pearson’s correlation was calculated using the corr.test function and the results were visualized using the Pheatmap package. The significance of each OTU-gene pair correlation was determined with a P-adjust threshold of <=0.05, ensuring that only robust and significant correlations were considered in the analysis.

### Validation of key genes and gut microbiome

We validated the key genes and gut microbiome identified in our study using publicly available databases. Gene expression data were sourced from TCGA (The Cancer Genome Atlas https://www.cancer.gov/tcga) and GSE117606 from GEO (Gene Expression Omnibus, https://www.ncbi.nlm.nih.gov/geo) (https://doi.org/10.1093/nar/gks1193), while gut microbiome data were obtained from the GMrepo database (https://gmrepo.humangut.info/) (Dai et al., 2022).

## Results

### Patient cohort characteristics

A total of 10 patients diagnosed with both CRC and synchronous polyps were enrolled in this study, comprising 5 males and 5 females, with a mean age of 69.6 ± 9.3 years. Tumor samples were collected from the rectum, sigmoid colon, and ascending colon, and all patients were diagnosed with moderately differentiated adenocarcinoma. The cancer staging was distributed as follows: 1 patient in stage I, 5 in stage II, and 4 in stage IV. The healthy control group comprised 13 participants, including 10 males and 3 females, with a mean age of 47.6 ± 10.3 years (see **Supplementary Table 1**).

### Differences in gut microbiota composition between CRC patients and healthy controls

**Figure 1:**
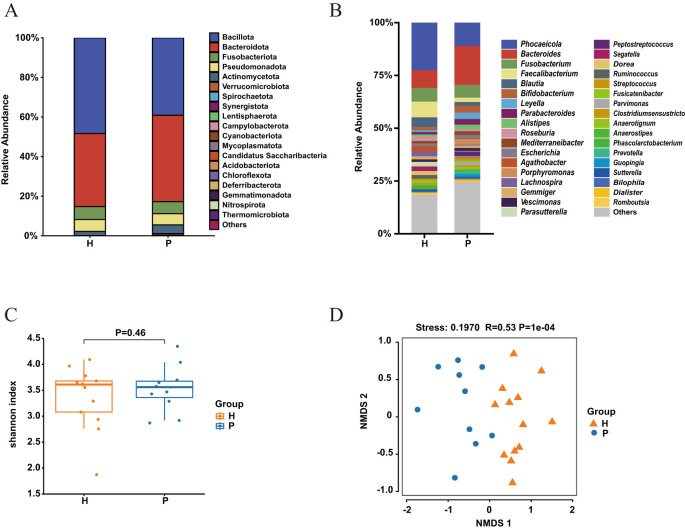
Gut bacterial composition and diversity in colorectal cancer (CRC) patients (P) and healthy controls (H). **(A)** Relative abundance at the phylum level and **(B)** genus level in CRC patients (blue) and healthy controls (yellow). **(C)** alpha-diversity assessed by the Shannon index for healthy controls (yellow) and CRC patients (blue), with p > 0.05 (T-test). **(D)** Non-metric multidimensional scaling (NMDS) of the 16S rRNA gene sequences from fecal samples in CRC patients (P) and healthy controls (H). The NMDS plot features a stress value of 0.1970 and a correlation coefficient (R) of 0.53, calculated using Bray-Curtis distances, with a highly significant P-value of 1e-04.

In both CRC patients and healthy controls, four dominant bacterial phyla—Firmicutes, Bacteroidetes, Fusobacteria, and Proteobacteria—accounted for 94% to 98% of the OTUs on average (**Figure 1A**). Compared to the healthy controls, the relative abundance of Bacteroidetes and Fusobacteria was significantly increased in CRC patients, while the relative abundance of Firmicutes was significantly decreased (P < 0.05) (**Figure 1B**). Alpha diversity, evaluated using the Shannon diversity index, indicated a trend toward reduced gut microbial diversity in CRC patients, although this trend was not statistically significant (**Figure 1C**). Beta diversity analysis, conducted using NMDS, demonstrated a significant difference in microbial community composition between CRC patients and healthy controls (P < 0.01) (**Figure 1D**).

### Identification of differentially abundant bacterial taxa

**Figure 2:**
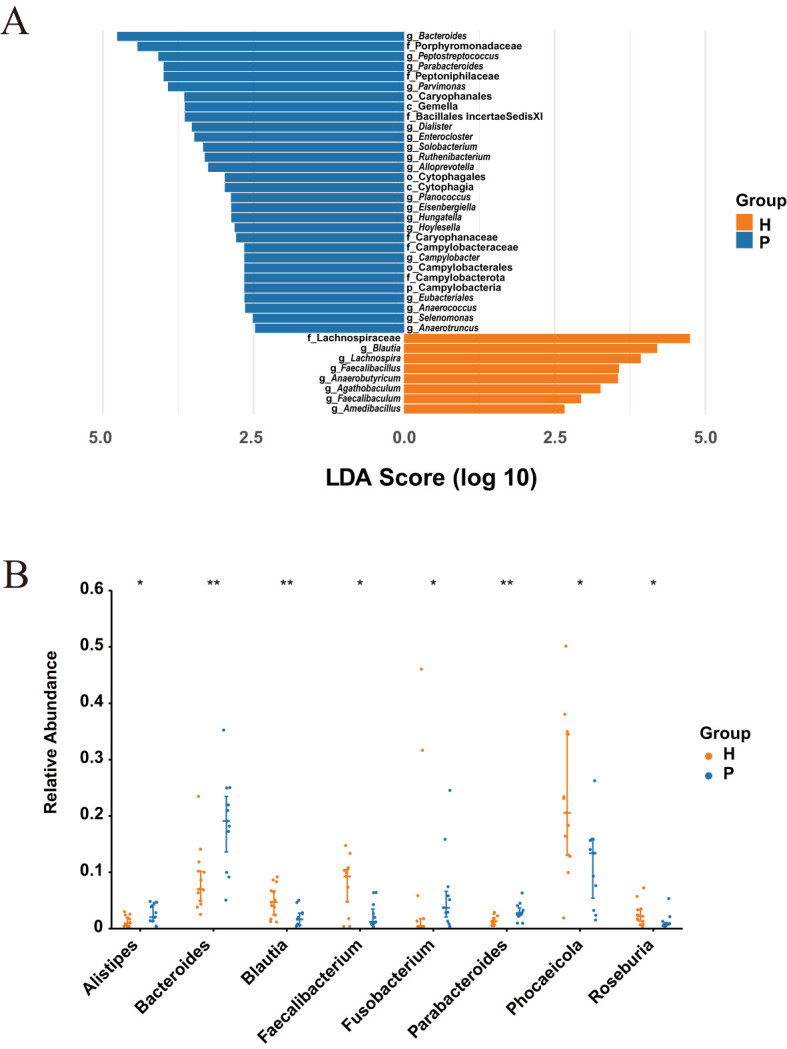
Differential abundance of microbiota between CRC patients (P) and healthy controls (H). **(A)** LEfSe analysis with an LDA score > 2 and P-value < 0.01 (Wilcoxon rank-sum test). **(B)** The top 8 differential genera in CRC patients (P) and healthy controls (H) with P-value < 0.05 (Wilcoxon rank-sum test). * indicates P-value < 0.05, ** indicates P-value < 0.01.

LEfSe analysis identified 38 significantly different taxa across five taxonomic levels (phylum, class, order, family, and genus) (**Figure 2A**). CRC patients showed significant enrichment of 30 taxa, including the genera *Bacteroides*, *Peptostreptococcus*, *Parabacteroides*, and the family Porphyromonadaceae. In contrast, healthy controls exhibited significant enrichment of 8 taxa, predominantly from the family Lachnospiraceae and genus *Blautia*. At the genus level, the Wilcoxon rank-sum test substantiated the differential abundance. The relative abundance of genera *Alistipes*, *Bacteroides*, and *Parabacteroides* was significantly higher in CRC patients (all P < 0.05), with *Bacteroides* and *Parabacteroides* showing highly significant differences (P < 0.01) (**Figure 2B**). In the healthy controls, the relative abundance of genera *Blautia*, *Faecalibacterium*, *Phocaeicola*, and *Roseburia* was significantly elevated (all P < 0.05).

### Differential gene expression across CRC tissues, normal mucosa, and polyp tissues

**Figure 3:**
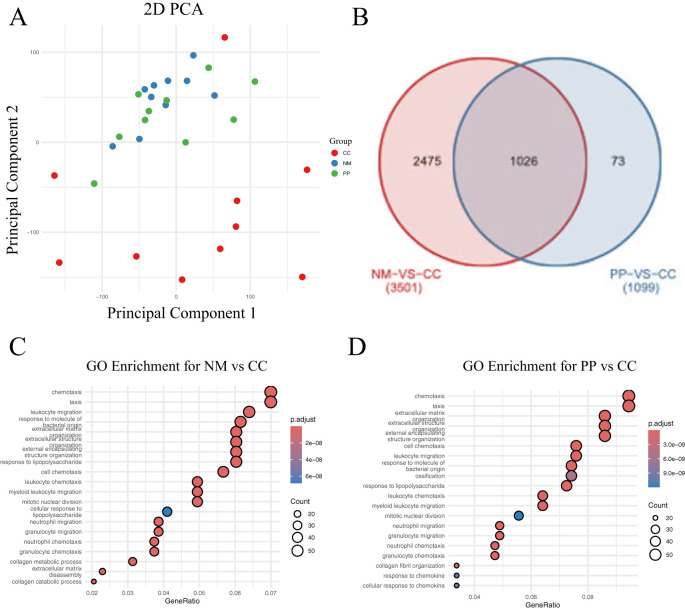
Gene expression analysis and GO enrichment in two comparisons. **(A)** The 2D plot shows the distribution of samples based on gene expression data. Points are colored to represent different groups: red for tumor, green for polyp, and blue for normal tissue. **(B)**. Venn diagram displaying the differentially expressed genes (DEGs) between the NM vs CC and PP vs CC comparisons. The overlapping region represents the common genes shared between both comparisons: tumor tissue (denoted as CC) versus normal mucosa (denoted as NM), and tumor tissue to colorectal polyps (denoted as PP). **(C)**. Top 20 GO enrichment for the NM vs CC comparison. **(D)**. Top 20 GO enrichment for the PP vs CC comparison. CC: tumor tissue, NM: normal mucosa, PP: colorectal polyps.

Transcriptome sequencing was performed on CC, NM, and PP samples from 10 CRC patients. The 2D PCA plot illustrates the gene expression disparities among CC, NM, and PP groups (**Figure 3A**). Notably, CC group exhibited a markedly distinct distribution in the principal component space when compared to both NM and PP groups, suggesting that the transcriptomic profiles of CC divergent from those of NM and PP. In the comparison between CC and NM, a total of 3,501 DEGs were identified, while 1,099 DEGs were identified between CC and PP (**Figure 3B**). After excluding genes unique to each comparison, 1,026 common DEGs were selected for further analysis.

GO analysis revealed a significant enrichment of genes related to bacterial molecular responses in CC tissue (**Figures 3C, D**). This finding suggests that the gut microbiota may play a crucial role in cancer tissues, with distinct differences in bacterial-related responses between cancerous and non-cancerous tissues. These findings bolster our understanding of the potential role of the gut microbiome in CRC pathogenesis and provides new insights for exploring the interplay between the gut microbiota and cancer development.

### Correlation analysis between gut microbiota and host gene expression

**Figure 4:**
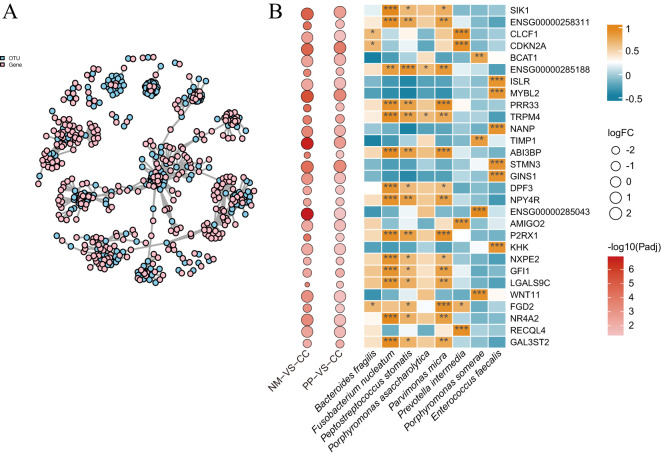
Correlation network and pearson correlation analysis. **(A)**. This network plot illustrates the correlations between OTUs and genes. The nodes in the plot represent OTUs and gene names, while the edges indicate the strength of the correlation, with edge thickness reflecting the magnitude of the correlation. OTU nodes are colored sky blue, and gene name nodes are colored pink. **(B)**. Pearson correlation coefficients of 100 OTU-gene pairs (P-adjust < 0.05). The intensity of the colors represents the degree of correlation; correlations were considered significant at P-adjust < 0.05. P-adjust < 0.001, P-adjust < 0.01, and P-adjust < 0.05 were labeled with “***”, “**” and “*” , respectively. The left panels denote the fold changes and P-adjust of DEGs in two groups (NM vs CC and PP vs CC). CC: tumor tissue, NM: normal mucosa, PP: colorectal polyps.

This study conducted Pearson correlation analysis between 1,026 DEGs and 298 OTUs, retaining data with adjusted P values (P-adjust) less than 0.05 for network visualization (**Figure 4A**). The network plot provided a clear view of the interactions between OTUs and genes, identifying 64 significant OTU-gene pairs, involving 29 mRNAs and 7 OTUs (**Figure 4B**). Within this set, 17 pairs had P-adjust <0.05, 17 pairs had P-adjust < 0.01, and 30 pairs had P-adjust < 0.001, with the majority of correlations being positive.

The study found that the genes tissue inhibitor of metalloproteinases 1 (*TIMP1*) and branched chain aminotransferase 1 (*BCAT1*) were significantly positively correlated (correlation coefficient r > 0.76, P < 0.01) with pathogenic bacteria such as *Fusobacterium nucleatum*, *Peptostreptococcus stomatis*, and *Parvimonas micra*. Additionally, the gene transient receptor potential cation channel subfamily M member 4 (*TRPM4*) showed significant correlations with multiple pathogenic bacteria. Other genes, including MYB proto-oncogene like 2 (*MYBL2*), cyclin dependent kinase inhibitor 2A (*CDKN2A*), and stathmin 3 (*STMN3*) were significantly upregulated in tumor tissues from CRC patients and were significantly associated with specific bacterial genera (**Figure 4B**).

### Validation of key gene expression and gut microbiome

The results show that among the eight bacteria, *Bacteroides* fragilis exhibits a particularly strong association with colon adenomas. Notably, the relative abundance of *Porphyromonas asaccharolytica* and *Porphyromonas somerae* is the highest among the analyzed species (**Supplementary Figure S1**).

**Figure 5:**
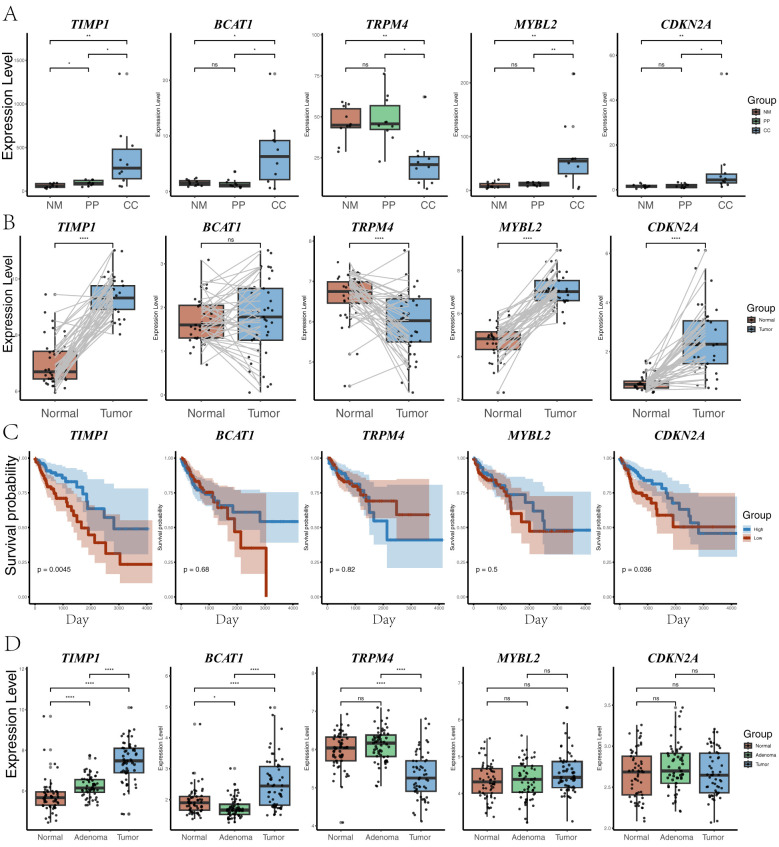
Gene expression analysis across different groups. **(A)** Expression levels of five genes (*TIMP1*, *BCAT1*, *TRPM4*, *MYBL2*, and *CDKN2A*) across three groups (CC: tumor tissue, NM: normal mucosa, PP: colorectal polyps). Statistical significance between the groups is indicated by P-value. P-value < 0.001, P-value < 0.001, P-value < 0.01, and P-value < 0.05 were labeled with “****”, “***”, “**” and “*” , respectively. **(B)** Distribution of the same five genes in TCGA paired samples between normal and tumor tissues, highlighting changes in gene expression between matched sample sets. **(C)** Survival analysis based on the expression levels of the five genes in TCGA samples. Kaplan-Meier plots show the relationship between high and low expression of each gene and overall survival probability. **(D)** Expression levels of the five genes (*TIMP1*, *BCAT1*, *TRPM4*, *MYBL2*, and *CDKN2A*) in the GSE117606 dataset, across normal, adenoma, and tumor groups.

Expression analysis of five key genes in CC, NM, and PP revealed that BCAT1, *MYBL2*, *CDKN2A*, and *TIMP1* were significantly upregulated in CC compared to NM and PP (all P < 0.05). Conversely, *TRPM4* was significantly downregulated in CC relative to NM and PP (P < 0.05). Analysis of TCGA database showed that *CDKN2A*, *MYBL2*, and *TIMP1* were significantly upregulated in cancer tissues, while *TRPM4* was significantly downregulated; *BCAT1* expression did not show a significant difference (**Figure 5**). GEO database analysis indicated that *TIMP1* and *BCAT1* were significantly upregulated in cancer tissues, whereas *TRPM4* was significantly downregulated, with no significant differences in *MYBL2* and *CDKN2A* expression. Additionally, TCGA data revealed that high expression of *CDKN2A* and *TIMP1* were significantly associated with poorer survival outcomes (P = 0.036 and P = 0.0045, respectively). In contrast, the expression of *TRPM4*, *BCAT1*, and *MYBL2* was not significantly correlated with survival (all P-values > 0.05).

## Discussion

In this study, we conducted a comprehensive analysis of patients with CRC accompanied by synchronous polyps, revealing alterations in gut microbiota composition and host gene expression, and their interrelationships. To our knowledge, this is the first study to collect CC, NM, and PP from the same patient, thereby eliminating inter-individual variability and more precisely reflecting the biological changes during disease progression. This approach contrasts sharply with previous studies that treated CRC patients, polyp patients, and healthy controls as separate groups, providing fresh insights into the mechanisms underlying CRC initiation and progression.

Our findings demonstrated significant changes in the gut microbiota composition of CRC patients with synchronous polyps, characterized by an increase in pathogenic bacteria and a decrease in beneficial bacteria. This result is consistent with previous studies, further validating the crucial role of gut microbiota in the pathogenesis of CRC (Liu et al., 2020; Silva et al., 2021). For instance, we observed a significant increase in the abundance of phyla the Bacteroidetes and Fusobacteria, and a significant decrease in Firmicutes in these patients. This aligns with previous findings, which reported decreased gut microbial diversity and dysbiosis in CRC patients (Yeoh et al., 2020; Shariati et al., 2021; Cui et al., 2022). However, regarding α-diversity, our study showed a decreasing trend in gut microbial diversity in CRC patients with synchronous polyps, but it did not reach statistical significance. This finding contrasts with other studies that reported a significant reduction in α-diversity among CRC patients (Gataullin et al., 2021; Zhao et al., 2021), which reported a significant reduction in α-diversity in CRC patients. The discrepancy may be attributed to our relatively small sample size, which could reduce the power of statistical tests.

Additionally, we confirmed the enrichment of *Fusobacterium nucleatum* in CRC patients with synchronous polyps, a bacterium associated with tumor invasiveness and poor prognosis, supporting its pivotal role in CRC progression (Xu et al., 2021a). *F. nucleatum* interacts with host E-cadherin through its adhesin FadA, activating the β-catenin signaling pathway and upregulating the oncogene *MYC* (Rubinstein et al., 2019). Additionally, it interacts with the TIGIT receptor on T cells via its surface protein Fap2, inhibiting the antitumor activities of natural killer cells and T cells, leading to immune evasion (Galaski et al., 2021). *F. nucleatum* can also induce the release of pro-inflammatory cytokines such as IL-6 and TNF-α, creating an inflammatory microenvironment that promotes tumor development (Bostanghadiri et al., 2023). Other pathogenic bacteria, such as *Parvimonas micra*, *Prevotella intermedia*, *Bacteroides fragilis*, and *Enterococcus faecalis*, are also believed to promote CRC through various mechanisms, consistent with previous studies (Lavoie et al., 2020; Kabwe et al., 2021; Osman et al., 2021; Lee et al., 2022). In contrast, beneficial bacteria enriched in the healthy control group, such as *Blautia*, *Faecalibacterium*, and *Roseburia*, typically exhibit anti-inflammatory effects and maintain intestinal barrier function (Mohebali et al., 2020; Xia et al., 2021). Reducing these beneficial microbes may lead to an imbalance in the intestinal microenvironment, thereby promoting tumorigenesis.

Abnormal host gene expression was also observed. Transcriptome analysis revealed that genes such as *TIMP1*, *BCAT1*, and *MYBL2* were significantly upregulated in CRC tissues compared to normal mucosa and polyp tissues. Previous study has confirmed that overexpression of *BCAT1* in CRC is associated with tumor progression and poor prognosis (Symonds et al., 2022). Our study further emphasizes the critical role of this gene in CRC. *BCAT1*, as a key enzyme in branched-chain amino acid metabolism, promotes tumor cell proliferation (Mao et al., 2021). The methylation status of *BCAT1* has also been used as a biomarker for non-invasive CRC diagnosis, highlighting its potential in early tumor detection (Xu et al., 2021b). Conversely, we observed that *TRPM4* was significantly downregulated in cancer tissues compared to normal mucosa and polyp tissues. This finding is consistent with analyses from TCGA and GEO databases, suggesting that TRPM4 may act as a tumor suppressor in CRC. However, a previous study indicated that *TRPM4* is highly expressed in tumor buds of human colorectal tumors and is associated with proliferation, cell cycle regulation, and invasion of colorectal cancer cells (Kappel et al., 2019). This contradiction may stem from differences in sample types, detection methods, or patient populations, warranting further investigation in future studies.

Furthermore, we found a significant correlation between the expression of *TIMP1* and the abundance of *Porphyromonas somerae*. TIMP1, as an endogenous inhibitor of matrix metalloproteinases, participates in the degradation and remodeling of the extracellular matrix (Guccini et al., 2021). TIMP1 predicts colon cancer progression and metastasis through the FAK-PI3K/AKT and MAPK pathways (Song et al., 2016). Our results show that *TIMP1* expression in CRC tissues is higher than in polyp and normal mucosa groups, validated in TCGA and GEO databases, and that high *TIMP1* expression is significantly associated with poorer survival outcomes. Functional enrichment analysis of high *TIMP1* expression suggests that *TIMP1* may influence tumor invasion and metastasis by regulating ECM remodeling, cell adhesion, cell migration, and related signaling pathways (such as ECM-receptor interaction and cytoskeleton regulation). *P. somerae*, a Gram-negative anaerobic bacterium belonging to the *Porphyromonas* genus, is commonly found in the oral microbiota and has been associated with various human diseases, particularly periodontitis and systemic inflammation (Lundmark et al., 2019). Study has found that in lean CRC, *P. somerae* is considered a characteristic microbe and is associated with functional metabolic pathways such as mucin O-glycan biosynthesis, glycosaminoglycan degradation, and butyrate metabolism (Zhu et al., 2024). Although direct studies linking *P. somerae* to CRC are relatively scarce, other species within the genus, such as *Porphyromonas gingivalis*, have been extensively studied and reported to play important roles in CRC pathogenesis (Wang et al., 2021; Kerdreux et al., 2023). *P. somerae* shares genomic similarities with *P. gingivalis*, including genes involved in energy metabolism and intracellular survival, suggesting that *P. somerae* may possess similar pathogenic mechanisms (Crooks et al., 2021). The gingipain virulence factors of *P. gingivalis* promote cell proliferation, inhibit apoptosis, and accelerate inflammatory responses by regulating the PI3K/AKT and MAPK signaling pathways, which are also involved in TIMP1-mediated colon cancer progression and metastasis (Liu et al., 2018). Additionally, gingipains can activate pro-inflammatory factors such as interleukins and tumor necrosis factors, further promoting tumor cell growth and invasion (Kerdreux et al., 2023). Regarding the impact of *P. gingivalis* on *TIMP1* expression, studies have shown that *P. gingivalis* infection can enhance the secretion of MMP-1 and TIMP1 in human periodontal ligament fibroblasts, leading to an imbalance in the MMP-1/TIMP1 ratio and further promoting tissue destruction (Herath et al., 2013). Based on the aforementioned studies on *P. gingivalis*, we speculate that *P. somerae* may influence CRC occurrence and progression through similar mechanisms. This provides a novel perspective for considering *P. somerae* or its related inflammatory pathways as potential therapeutic targets for CRC. However, further research is necessary to elucidate the specific molecular mechanisms by which *P. somerae* affects *TIMP1* expression and to verify its role in CRC pathogenesis.

Additionally, increasing evidence suggests that *P. somerae* can modulate other key genes via metabolic pathways. For instance, Crooks demonstrated in an endometrial cancer model that *P. somerae* can invade epithelial cells, evade immune recognition, and secrete metabolic intermediates such as succinate, which stabilize hypoxia-inducible factors (HIF) (Crooks et al., 2021). Through this process, *P. somerae* promotes carcinogenesis by inducing chronic inflammation and enhancing angiogenesis. With respect to *BCAT1*, branched-chain aminotransferases (*BCAT*) are pivotal in the catabolism of branched-chain amino acids (BCAAs), and *BCAT1* is the predominant isoform in human primary macrophages (Papathanassiu et al., 2017). Notably, *P. somerae* often exhibits esterase, esterase lipase, leucine arylamidase, and valine arylamidase activities (Summanen et al., 2005), the latter two being able to hydrolyze aromatic amine compounds containing leucine or valine, thereby supplying additional leucine or valine substrates to the host. Under these conditions, the demand for *BCAT* by host cells likely increases, potentially resulting in *BCAT1* upregulation to facilitate BCAA transamination. Hence, we hypothesize that *P. somerae* indirectly induces *BCAT1* overexpression by altering the metabolic milieu or providing surplus substrates, in line with our observed positive correlation between *P. somerae* abundance and *BCAT1* expression. Further *in vitro* and *in vivo* investigations are warranted to validate this hypothesis and elucidate the precise molecular mechanisms involved.

Our study’s findings have potential clinical significance. First, our innovative study design, comparing different pathological tissues within the same patient, avoids inter-individual differences and more accurately reflects the biological changes during disease progression. Second, detecting specific pathogenic bacteria such as *P. somerae* may serve as biomarkers for early diagnosis or risk assessment of CRC. Interventions targeting these pathogens, or their associated pathways could provide new strategies for CRC prevention and treatment. For example, probiotic therapies that modulate the gut microbiota or antimicrobial treatments targeting specific bacterial populations may influence host gene expression and slow tumor progression. Finally, expression levels of genes like *TIMP1* could serve as reference indicators for prognostic evaluation and personalized treatment.

Despite providing important insights, our study has several limitations. First, the sample size is relatively small, including only 10 CRC patients, which may limit the generalizability of the results. However, our findings are consistent with mainstream research, supporting the reliability of our conclusions. Second, the study primarily focuses on correlational analyses and lacks in-depth exploration of the causal relationships between microbiota and gene expression. Future studies should incorporate *in vivo* and *in vitro* experiments to investigate the specific mechanisms by which particular microbes affect host gene expression. Additionally, due to the absence of longitudinal data, we could not assess the dynamic changes of microbiota and gene expression during disease progression. Future longitudinal studies will help elucidate the roles of these changes in CRC development and progression.

## Conclusion

Through a pioneering study design, our research conducted an in-depth analysis of the alteration in gut microbiota and host gene expression in CRC patients with synchronous polyps. We revealed, for the first time, a significant association between the abundance of *P. somerae* and the high expression of *TIMP1*. These findings shed new light on the mechanisms underlying the onset and progression of CRC and suggest potential avenues for early diagnosis, prevention, and the development of personalized treatment strategies.

## Data Availability

Publicly available datasets were utilized in this study as follows: Gene expression data were obtained from the TCGA (The Cancer Genome Atlas, https://www.cancer.gov/tcga) and the GSE117606 dataset from GEO (Gene Expression Omnibus, https://www.ncbi.nlm.nih.gov/geo, https://doi.org/10.1093/nar/gks1193). Gut microbiome reference data were accessed from the GMrepo database (https://gmrepo.humangut.info/). The data generated and analyzed during the current study are available from the corresponding author upon reasonable request.
