# Supplementary material for: Comprehensive analysis of transcriptome and microbiome in colorectal cancer with synchronous polyp patients
Source: Front Cell Infect Microbiol. 2025 Apr 17;15:1547057. doi: 10.3389/fcimb.2025.1547057 (PMC12043645; doi:10.3389/fcimb.2025.1547057)
Supplement: Supplementary file 1 [file DataSheet1.docx]

**Figure S: The Abundance Distribution of Bacteria Across Diverse Phenotypes**

The distribution of abundance medians for eight bacteria, including *Bacteroides fragilis, Fusobacterium nucleatum, Peptostreptococcus stomatis, Parvimonas micra, Prevotella intermedia, Porphyromonas somerae, Enterococcus faecalis,* and *Porphyromonas asaccharolytica,* across various phenotypes, is presented. Each bar represents a specific phenotype. The x-axis displays the median abundance of the corresponding species, while the y-axis lists the phenotype categories. Each bar is annotated with a label indicating the number of valid runs in which the species was found (X), the total number of valid runs for that phenotype (Y).
