# Supplementary material for: Comprehensive analysis of transcriptome and microbiome in colorectal cancer with synchronous polyp patients
Source: Front Cell Infect Microbiol. 2025 Apr 17;15:1547057. doi: 10.3389/fcimb.2025.1547057 (PMC12043645; doi:10.3389/fcimb.2025.1547057)
Supplement: Supplementary file 2 [file Table1.docx]

Sup.Tab 1：Clinical Characteristics of Colorectal Cancer Patients with Synchronous Polyps and Healthy Controls

| **Group** | **ID** | **Gender** | **Age** | **Location** | **Pathology** | **Stage** | |
| --- | --- | --- | --- | --- | --- | --- | --- |
| Patient | 1 | Male | 57 | Sigmoid Colon | Adenocarcinoma  (Moderately Differentiated) | IVB (cTxNxM1b) |  |
| Patient | 2 | Male | 77 | Ascending Colon | Adenocarcinoma  (Moderately Differentiated) | IIA (pT3N0M0) |  |
| Patient | 3 | Female | 70 | Rectum | Adenocarcinoma  (Moderately Differentiated) | IIA (pT3N0M0) |  |
| Patient | 4 | Female | 78 | Rectum | Adenocarcinoma  (Moderately Differentiated) | IIA (pT3N0M0) |  |
| Patient | 5 | Male | 76 | Rectum | Adenocarcinoma  (Moderately Differentiated) | I (pT2N0M0) |  |
| Patient | 7 | Female | 85 | Rectum | Adenocarcinoma  (Moderately Differentiated) | IVA (pT4bNxM1a) |  |
| Patient | 8 | Female | 57 | Sigmoid Colon | Adenocarcinoma  (Moderately Differentiated) | IV (cT3N2M1) |  |
| Patient | 9 | Male | 73 | Sigmoid Colon | Adenocarcinoma  (Moderately Differentiated) | IIA (cT3N0M0) |  |
| Patient | 10 | Male | 70 | Rectum | Adenocarcinoma  (Moderately Differentiated) | IIA (cT3N0M0) |  |
| Patient | 11 | Female | 53 | Sigmoid Colon | Adenocarcinoma  (Moderately Differentiated) | IVA (cT4bN1aM1) |  |
| Control | 1 | Male | 50 | - | - | - |  |
| Control | 2 | Female | 27 | - | - | - |  |
| Control | 3 | Male | 63 | - | - | - |  |
| Control | 4 | Female | 57 | - | - | - |  |
| Control | 5 | Male | 52 | - | - | - |  |
| Control | 6 | Male | 49 | - | - | - |  |
| Control | 7 | Male | 30 | - | - | - |  |
| Control | 8 | Male | 39 | - | - | - |  |
| Control | 9 | Female | 44 | - | - | - |  |
| Control | 10 | Male | 50 | - | - | - |  |
| Control | 11 | Male | 45 | - | - | - |  |
| Control | 12 | Male | 45 | - | - | - |  |
| Control | 13 | Male | 59 | - | - | - |  |
